# Improving sentiment classification using a RoBERTa-based hybrid model

**DOI:** 10.3389/fnhum.2023.1292010

**Published:** 2023-12-07

**Authors:** Noura A. Semary, Wesam Ahmed, Khalid Amin, Paweł Pławiak, Mohamed Hammad

**Affiliations:** ^1^Department of Information Technology, Faculty of Computers and Information, Menoufia University, Shibin El Kom, Egypt; ^2^Department of Information Technology, Faculty of Computers and Artificial Intelligence, South Valley University, Hurghada, Egypt; ^3^Department of Computer Science, Faculty of Computer Science and Telecommunications, Cracow University of Technology, Krakow, Poland; ^4^Institute of Theoretical and Applied Informatics, Polish Academy of Sciences, Gliwice, Poland; ^5^EIAS Data Science Lab, College of Computer and Information Sciences, Prince Sultan University, Riyadh, Saudi Arabia

**Keywords:** sentiment analysis, word embedding, RoBERTa, SMOTE, LSTM, CNN+LSTM

## Abstract

**Introduction:**

Several attempts have been made to enhance text-based sentiment analysis’s performance. The classifiers and word embedding models have been among the most prominent attempts. This work aims to develop a hybrid deep learning approach that combines the advantages of transformer models and sequence models with the elimination of sequence models’ shortcomings.

**Methods:**

In this paper, we present a hybrid model based on the transformer model and deep learning models to enhance sentiment classification process. Robustly optimized BERT (RoBERTa) was selected for the representative vectors of the input sentences and the Long Short-Term Memory (LSTM) model in conjunction with the Convolutional Neural Networks (CNN) model was used to improve the suggested model’s ability to comprehend the semantics and context of each input sentence. We tested the proposed model with two datasets with different topics. The first dataset is a Twitter review of US airlines and the second is the IMDb movie reviews dataset. We propose using word embeddings in conjunction with the SMOTE technique to overcome the challenge of imbalanced classes of the Twitter dataset.

**Results:**

With an accuracy of 96.28% on the IMDb reviews dataset and 94.2% on the Twitter reviews dataset, the hybrid model that has been suggested outperforms the standard methods.

**Discussion:**

It is clear from these results that the proposed hybrid RoBERTa–(CNN+ LSTM) method is an effective model in sentiment classification.

## 1 Introduction

Sentiment analysis (SA) is the examination of a person’s attitudes, feelings, and sentiments as expressed in their writing (Goodrum et al., 2020). It is among the most significant areas within the field of natural language processing (NLP). These days, sentiment analysis is widespread in social media and business. Social media’s explosive growth has made it possible for anyone to voice their thoughts and opinions online. Thus, sentiment analysis is essential to understanding what customers or reviewers think about. In addition, sentiment analysis is an effective tool for examining the public’s response to political issues (Vadivukarassi et al., 2018; Birjali et al., 2021). Text representation, which is used to translate text data into a numerical form that computers can understand, is an essential part of the sentiment analysis process (Khan and Yairi, 2018). Word embeddings are an essential part of many natural language processing (NLP) applications because they help with information extraction tasks, which involve extracting relevant data from unstructured text, and text clustering, which involves grouping related documents together based on their content. Word embeddings are useful for tasks like sentiment analysis and document classification. Words can be aggregated to produce a representation at the document level by being represented as vectors. Then, for tasks like document classification (assigning a category or topic to a document) or sentiment analysis (figuring out the sentiment expressed in a text), this representation can be fed into a machine-learning or deep learning model. Several limitations and considerations to take into account when employing word embeddings are emphasized by the challenges. Contextual information is not captured by word embeddings, which are static representations. The surrounding context, however, can change a word’s meaning. Word embeddings that take into account the context in which a word appears like ROBERTa, BERT, or GPT have been developed as a solution to this limitation and can help capture the nuanced expressions of sentiment within the context of the text. Vectors with a high dimension are usually word embeddings. The intricacy and computational demands of downstream models may rise when dealing with large embedding dimensions.

Various methods have been suggested to capture features from the text. The transformer model, which utilizes the attention mechanism, is one of the most widely used feature representations and has demonstrated exceptional results in NLP lately (Ahmad and Wu, 2023). Recently, the highest level of performance has been shown by many transformer models for various NLP tasks, including BERT (Bidirectional Encoder Representations from Transformers), ALBERT (A Lite BERT For Self-Supervised Learning Language Representations), and RoBERTa (Robustly Optimized BERT Pre-training Approach). These models can selectively weigh distinct segments of the input sequence to generate informative embeddings through the attention mechanism. More specifically, the attention mechanism calculates the input embeddings’ weighted sum where the weights are chosen by a compatibility function that is learned between the query and the key embeddings (Liu et al., 2019). Consequently, long-range dependencies in the input sequence can be captured by the model, improving its ability to represent the input. For sentiment analysis, several deep learning (DL) and machine learning (ML) methods were proposed, especially sequence models that could encode the text’s long-distance dependencies. Many different applications, including energy and medicine, use Recurrent Neural Network (RNN) -based modeling techniques (Deng and Yu, 2014; Choudhary et al., 2022). However, when processing is serialized, the sequence models are less computationally efficient (Dholpuria et al., 2018). On the other hand, by employing parallelized processing, the transformer models enhance computation. As a result, the hybrid deep learning model that combines the advantages of sequence models and transformer models is proposed and this study investigated how hybrid models affected the accuracy of the results in sentiment classification.

Contributions to our work include the following:

(1) A pre-trained RoBERTa feature extraction model is proposed for the extraction of text and aspect token features. Since the pre-trained RoBERTa model is trained on a large number of corpora, its performance will be stable.
(2) The SMOTE technique in the Twitter reviews dataset is employed in conjunction with word embedding training to create samples that are lexically diverse and oversample minorities. This improves the model’s generalization ability, and a solution is found to the imbalanced dataset issue by providing more lexically rich training samples.
(3) For dataset classification, our hybrid deep learning model combines convolutional neural networks (CNN) and long short-term memory (LSTM), and we tuned hyperparameters to determine which settings yield the best classification results.
(4) Providing an analysis of how the hybrid deep learning algorithm with the RoBERTa word embedding model performs better than the most recent models for capturing sentiment from social media and accomplishing great results.

The remaining sections of the study are arranged as follows: The more recent sentiment analysis studies are presented in section 2. Section 3 explains the proposed work’s methodology. The results are shown in Section 4. The findings and constraints are covered in section 5. Section 6 contains the conclusion and future work.

## 2 Background literature

The purpose of this section is to review relevant studies on sentiment classification. The approaches can be separated into two primary groups: deep learning methods and machine learning methods.

### 2.1 Previous studies

The first and most important part of the text classification task is word embedding. Most of the feature extraction algorithms used in sentiment analysis have been developed using ML or DL models. The extracted features will be more instructive for the learning algorithm if you select the appropriate feature extraction technique, which will guarantee that the features capture relevant details and patterns in the data. This enhances the algorithm’s capacity for generalization and making accurate predictions. Some works have substituted the RoBERTa transformer-based model for text representation models. When RoBERTa model is pre-trained on a large corpus, it achieves an impressive outcome when compared to earlier NLP models. As shown in Table 1, sentiment analysis has been compared with other methods.

**TABLE 1:** An overview of the research on sentiment analysis.

References	Dataset	Word embedding	Model	Result
Tan et al., 2023	Twitter US Airline, Sentiment140, and IMDB reviews	RoBERTa	GRU	Accuracy = 89.59% in Sentiment140, 91.52% in Twitter, and 94.63% in IMDb
Tan et al., 2022	IMDB reviews, Sentiment140, and Twitter US Airline	RoBERTa	LSTM	Accuracy = 92.96% in IMDb, 91.37% in Twitter, and 89.70% in Sentiment140
Jang et al., 2020	IMDB movie	Word2Vec	MLP, CNN, LSTM, and Bi-LSTM+CNN	Bi-LSTM+CNN achieves high accuracy = 91.41%
Saad, 2020	Twitter US Airline	BOW	SVM, LR, and RF	SVM has the highest accuracy = 83.31%
Umer et al., 2021	Hate speech, Twitter US Airlines, and women’s e-commerce clothing	TF-IDF and Word2Vec	(CNN+LSTM) LR, VC, LR, and RF	CNN+LSTM has the highest accuracy = 92% in hate speech, 82% in Twitter, and 78 % in women’s e-commerce clothing,
Kumar et al., 2020	Book reviews	BOW, and Word2Vec	NB, ME, and SVM	SVM has the highest accuracy = 78%
Rahat et al., 2019	Twitter US Airlines sentiment	TF-IDF	SVM and NB	SVM outperforms NB and accuracy = 82.48%
Prabhakar et al., 2019	Twitter US Airlines sentiment	TF-IDF	SVM, DT, RF, Boosting and Adaboost	Adaboost approach outperforms others and the accuracy = 78%

Tan et al. (2023) suggested a novel hybrid sentiment analysis model. The suggested model employs the Gated Recurrent Units (GRU) and Robustly Optimized BERT Pretraining Approach (RoBERTa) models. The model was evaluated using three widely used sentiment analysis datasets: Twitter US reviews, Sentiment140, and IMDB. According to the findings, the model’s accuracy on Sentiment140 (89.59%), Twitter US Airline Sentiment (91.52%), and IMDB (94.63%).

Tan et al. (2022) used a new approach that combines the transformer model with the deep learning model. This paper used the RoBERTa model with the Long Short-Term Memory model (LSTM). With F1 scores of 93%, 91%, and 90% on the Sentiment 140, IMDb, and Twitter reviews datasets, respectively, the suggested hybrid model performs better than different techniques.

Jang et al. (2020) suggested a Bi-LSTM and CNN hybrid model that incorporates an additional attention mechanism to leverage the benefits of both LSTM and convolutional neural network (CNN). IMDB movie review data was used to train the model. The hybrid attention Bi-LSTM and CNN model exceeds individual LSTM, CNN, and multi-layer perceptron (MLP) models with regard to accuracy F1 scores, recall, and accuracy. The suggested hybrid attention achieves accuracy (91.41%).

Saad (2020) used some ML algorithms with a Bag of Words (BoW) feature extraction method on the Twitter review sentiment dataset. To classify tweets, there are several options: Naïve Bayes (NB), Support Vector Machine (SVM), Logistic Regression (LR), XgBoost (XGB), Random Forest (RF), and Decision Tree (DT). The performance is determined by the Precision, Accuracy, F1-score, and Recall for every classifier. The SVM model had the highest accuracy (83.31%).

Umer et al. (2021) introduced a combination of CNN and LSTM models (CNN-LSTM) using three datasets (hate speech, Twitter reviews, and women’s e-commerce clothing). The RF, stochastic gradient descent (SGD), a voting classifier (VC) of (RF- SGD), LR, and SVM are among the ML models that are used to assess how well the proposed model performs. In addition, on prediction accuracy, the effects of two feature extraction methods [word2vec and term frequency–inverse document frequency (TF-IDF)] are also investigated. The experiment’s findings demonstrate that the accuracy achieved by the CNN-LSTM is higher than that of the other classifiers with 82% in the Twitter US dataset.

Kumar et al. (2020) the impact of age and gender on the collected customer reviews was studied. LSTM, SVM, and maximum entropy (ME) models are used. Word2vec is used in the LSTM model, whereas the BOW feature extraction is used by the SVM, ME, and NB algorithms. For female data, the group over 50 has the best accuracy.

Rahat et al. (2019) examined some machine learning algorithms using Twitter review sentiment. This paper’s main objective is to compare the overall accuracy, precession, and recall values of the NB and SVM algorithms. The outcome demonstrates that SVM was the most effective and produced the highest accuracy (82.48%) in the case of airline reviews.

Prabhakar et al. (2019) used a new approach in a machine learning algorithm based on the adaboost Approach to classify the Twitter US Airlines sentiment dataset. Some ML algorithms have been applied. Based on performance metrics including recall, accuracy, and precision, the proposed model’s results showed improved accuracy (78%).

### 2.2 Gap in literature

Most of the examined studies use different feature extraction techniques but there are limited studies that use transformer models for text embedding, which is surprising since text embedding is an essential component of sentiment analysis. The extracted features will be more instructive for the learning algorithm if you select the appropriate feature extraction technique, which will guarantee that the features capture relevant details and patterns in the data. This enhances the algorithm’s capacity for generalization and making accurate predictions. Text embedding with transformers can be highly expressive, capture contextual relationships, and produce more accurate representations than traditional embedding techniques. Most studies employed hybrid models, which combined CNNs with LSTM or Bi-LSTM architecture. In this paper, we will combine the RoBERTa model with the hybrid deep learning model (CNN+LSTM).

## 3 Proposed system

**FIGURE 1:**
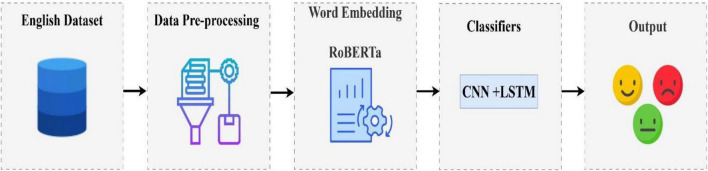
The suggested structure for sentiment classification.

The purpose of this section is to provide a description of the datasets, the preprocessing step, the word embedding method, the SMOTE technique, and the proposed classification model. In Figure 1, we show the framework of our proposed model.

### 3.1 Dataset description

**FIGURE 2:**
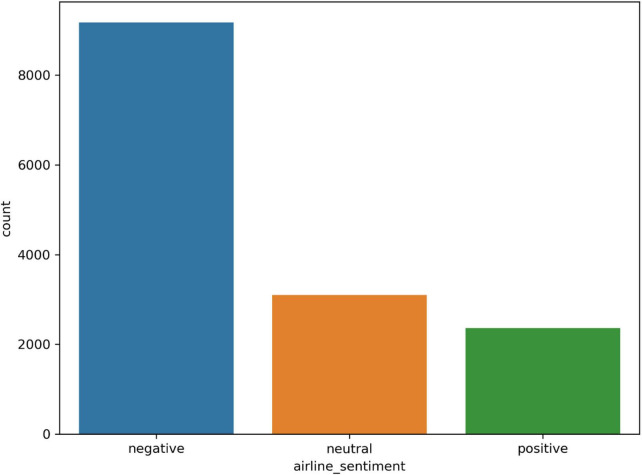
The Twitter dataset’s distribution.

**FIGURE 3:**
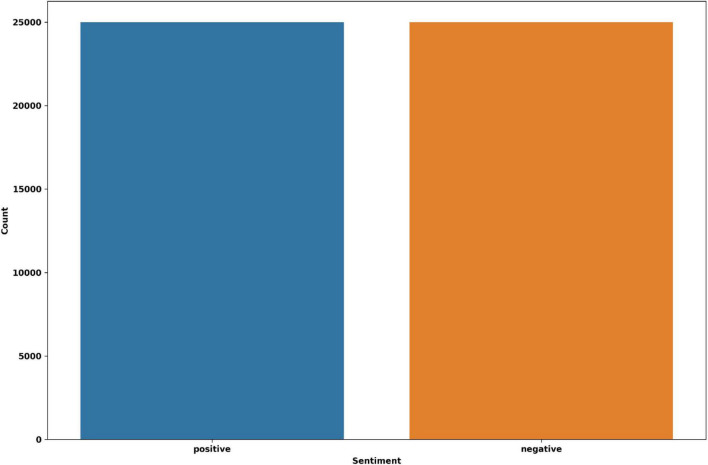
The IMDB dataset’s distribution.

We selected two distinct datasets for the experiments that include real-world consumer feedback. Twitter US Airlines is the first dataset (Habbat et al., 2023). It provides a collection of passenger feedback from six major American airlines, and it is imbalanced data. The total size is 14,640, of which 2,363 are positive, 9,178 are negative, and 3,099 are neutral tweets. The IMDB movie reviews are the second dataset, which contains balanced data (Tan et al., 2022). There are 50,000 reviews in total, of which 25,000 reviews are positive and 25,000 are negative. The distribution of sentiment classes in the datasets is shown in Figures 2, 3.

### 3.2 Text pre-processing

In the sentiment analysis procedure, the preprocessing step is crucial. This step converts text into a format that deep learning algorithms can understand (Chong et al., 2014). Retweets are eliminated as part of the text preprocessing since they might affect word frequency and increase the amount of space required to conduct the experiments. URLs should be eliminated in the following stage since they are meaningless and won’t impact sentiment. The tokenization step divides a text or paragraph into smaller pieces called tokens; therefore, it is important to remove punctuation, non-alphanumeric characters, emojis, and stop words as they are not useful for analysis. By taking this step, the complexity of the data is decreased, and word-level information is isolated (Sun et al., 2015; Chopra et al., 2022). Lastly, the stemming process reduces words to word stems because some of the words may not be appropriate in the language, and the lemmatization process eliminates inflectional endings and returns the base or dictionary form of words (Bordoloi and Biswas, 2023). In this study, PorterStemmer stemming and WordNetLemmatizer lemmatization were employed. Table 2 displays a few instances from IMDB reviews both before and after the preprocessing step.

**TABLE 2:** Some samples of IMDB reviews dataset.

Movie reviews	Movie reviews after pre-processing
One of the other reviewers has mentioned that…	One reviewers mentioned watching oz episode yo…
A wonderful little production. < br / > < br / > The…	wonderful little production br br filming tech…
I thought this was a wonderful way to spend ti…	thought wonderful way spend time hot summer we…
Basically there’s a family where a little boy…	Basically there’s family little boy jake thinks…

### 3.3 RoBERTa

Robustly Optimized BERT Pretraining Approach is an extended version of the BERT model. It stands for A Robustly Optimized BERT Pretraining Approach. The transformers family includes RoBERTa and BERT (Liao et al., 2021). To handle long-range dependencies in sequence-to-sequence modeling, transformer models are utilized. Heads, transformers, and tokenizers are the three parts of a transformer model. Raw text is transformed into sparse index encodings by the tokenizer. The transformers part then transforms sparse content into contextual embeddings for deeper learning (Narayanaswamy, 2021). To use contextual embedding for downstream processing, the heads wrap the transformers model. Many benefits come with this model. By using byte-level byte pair encoding, RoBERTa tokenizes with a smaller vocabulary and requires fewer computational resources. Additionally, the model’s dynamic masking allows it to learn from various input sequences, where the input sequences are duplicated while attention masks are applied (Sirisha and Bolem, 2022). Four different corpora were used to train RoBERTa.

### 3.4 Synthetic minority over-sampling technique

When data are insufficient, oversampling is employed. By increasing the sample size of rare observations, the dataset is attempted to be balanced. This study found an imbalance between positive, neutral, and negative polarities in the Twitter dataset. As a result of this imbalanced data, deep learning models may perform poorly since the decision surface may tilt in favor of the majority class (Maciejewski and Stefanowski, 2011). The imbalanced data may have a significant negative impact on the deep learning models’ performance because it may tilt the decision surface in favor of the majority class. This study uses synthetic minority oversampling (SMOTE) as the oversampling method. SMOTE is the state-of-the-art procedure proposed by Chawla et al. (2002). In addition to preventing information loss, this method is straightforward to interpret and integrate and helps to solve the overfitting problem for unbalanced datasets. By providing more examples of the minority class, oversampling aids in the improvement of the learning process. As a result, the algorithm can investigate a larger variety of patterns and provide the underrepresented class with more informed choices. Randomly, SMOTE finds the K-nearest neighbors of the smaller classes. A new minority class is constructed using K-nearest neighbors for each selected sample (Bunkhumpornpat et al., 2009). In order to improve sentiment analysis performance, the SMOTE technique is then applied to the Twitter dataset after the preprocessing step to enhance the representation of minority classes.

### 3.5 Hybrid deep learning model

The problem of vanishing gradients is handled by an LSTM, a kind of RNN (Sherstinsky, 2020). It stands for long short-term memory. Three gates (one input, one forgets, and one output) along with a memory cell form this structure. The gradients remain unchanged when the input and output gates are turned off and the forget gate is activated. This allows LSTMs to learn long-term relationships and minimize vanishing gradient issues. In text classification, CNN models have proven to be effective. CNNs are a special type of neural network that is commonly used in image processing (Phan et al., 2022). Convolutional neural networks are also known as CNNs. CNN uses convolutional layers to associate a subset of input with its preceding layers, which is why CNN layers are called feature maps. A polling layer is used in the CNN model to reduce the complexity of computations. The flattened layer is used to feed the polling layer’s output and map it to the next layer. In CNN, the final layer is usually fully connected. In our study, both the LSTM and CNN deep learning models are combined because they perform well on sentiment analysis (Iparraguirre-Villanueva et al., 2023). CNN is used to extract local features, while LSTM is used to capture long-distance dependencies. Both models are combined into one hybrid model (CNN-LSTM). The final step is to apply the SoftMax function to the classification layer.

### 3.6 RoBERTa-(CNN+LSTM)

To perform sentiment classification, the suggested RoBERTa-(CNN+LSTM) model combines the advantages of LSTM and CNN networks with the pre-trained transformer-based language model RoBERTa. Tokenizing the cleaned text into sub words or words is the first step in the proposed RoBERTa-(CNN+LSTM) model. In this study, we use the RoBERTa tokenizer. In RoBERTa, it includes some special tokens such as those (< s > and < /s >) for indicating the beginning and end of sentences, and the pad token for padding a word vector to reach its maximum length. Text is divided into sub words using the byte-level Byte-Pair Encoding (BPE) tokenizer in the RoBERTa model. The frequently used words won’t be divided by this tokenizer. Nevertheless, uncommon words will be divided into sub words (Joshy and Sundar, 2022). Using input ids and an attention mask, the RoBERTa tokenizer encodes the raw text. The numerical representation of the token and its indices are represented by the input ids. In contrast, the sequence is batch-assembled using the attention mask as an optional argument. Which tokens should and shouldn’t be attended to is indicated by the attention mask. Input ids are passed into RoBERTa base model as well as attention masks. The RoBERTa architecture has 125 million parameters, 12 base layers, and 768 hidden state vectors. In order to make it easier for the subsequent layers to extract the relevant information from the word embedding, the objective of the RoBERTa base layers is to generate a significant word embedding as the feature representation. The word embeddings from RoBERTa are then passed through a CNN which is the first layer of the hybrid suggested model. The CNN model applies a set of filters with a kernel size = 3 to capture local patterns and features within the sentence. The filters slide over the sequence of word embeddings, extracting features by performing convolutions. The output of the first layer is then passed into LSTM. The LSTM allows the model to capture both forward and backward dependencies in the sequence of features. The LSTM processes the sequence of features, considering the temporal dependencies and capturing the long-range contextual information in the sentence (Ullah et al., 2022). The final hidden state of the LSTM represents a summary of the sentence’s context and captures the sentiment-related information. This hidden state is passed through a fully connected layer, which maps the LSTM output to sentiment labels. The activation function SoftMax is used in the classification layer to generate the sentiment analysis dataset’s probabilistic class distribution.

### 3.7 Performance measures

We applied various common performance metrics to evaluate how well the suggested model performs. We specifically applied F1-measure, recall, accuracy, and precision (Al Amrani et al., 2018; Zarisfi Kermani et al., 2020). A confusion matrix can be used to illustrate the DL model and generate all four metrics. FN (False Negative), TN (True Negative), TP (True Positive), and FP (False Positive) are the components of this matrix (Barbounaki et al., 2021). The following formulas are implemented to assess classifier performance:


(1) $$\mathrm{Accuracy}:\frac{\text{TP}+\text{TN}}{\text{TP}+\text{TN}+\text{FP}+\text{FN}}$$

(2) $$\mathrm{Precision}:\frac{\text{TP}}{\mathrm{TP}+\text{FP}}$$

(3) $$\mathrm{Recall}:\frac{\text{TP}}{\mathrm{TP}+\text{FN}}$$

(4) $$\text{F}1-\text{Measure}:\text{ 2}*\frac{\text{Precision}*\text{ Recall}}{\text{Precision + Recall}}$$

## 4 Experimental results

We conducted our experiments using Google Colab, a GPU-based cloud platform offered by Google Inc. We applied a hybrid DL model using the RoBERTa word representation model to a set of datasets commonly used in sentiment analysis. The Twitter dataset has imbalanced sentiment classes, therefore SMOTE is applied. For all experiments, training, validation, and testing datasets are split into 60:20:20. To find the parameter values that produce the best results, hyperparameter tuning is carried out. Table 3 illustrates the hyper-parameters that we tested to determine which ones were best for running our proposed model. Various metrics are used to evaluate the proposed model, including recall, F1-score, accuracy, and precision. In these experiments, ML and DL sentiment analysis methods are comprehensively compared. Among the ML techniques are SVM (Rahat et al., 2019; Steinke et al., 2022), DT (Steinke et al., 2022), and KNN (Rahat et al., 2019). LSTM (Jang et al., 2020; Umer et al., 2021) CNN-LSTM (Jang et al., 2020; Umer et al., 2021), RoBERTa-LSTM (Tan et al., 2022), and RoBERTa-GRU (Tan et al., 2023) are a few examples of deep learning techniques. Table 4 compares all approaches with the suggested RoBERTa-(CNN-LSTM) model using the Twitter dataset. The suggested model exceeds the alternative techniques in the Twitter dataset and attains the highest accuracy.

**TABLE 3:** The RoBERTa-(CNN-LSTM) model’s hyperparameters.

Hyperparameter	Optimal value
CNN unit	256
LSTM unit	256
Optimizer	Adam
Epoch	25
Batch size	32
Dropout	0.4
Learning rate	0.00001

**TABLE 4:** The proposed model’s performance using a Twitter dataset.

Methods	Accuracy	Precision	Recall	F1-measure
NB	76.56	89.00	83.75	86.37
SVM	82.48	90.33	81.79	85.85
LSTM	76	81	78	79
CNN-LSTM	82	85	81	83
RoBERTa-LSTM	91.37	91	91	91
RoBERTa-GRU	91.52	91	91	91
Proposed model	94.2	94	94	93

On the IMDB reviews dataset, the suggested model and other techniques are contrasted in Table 5. The values of F1-measure, precision, accuracy, and recall are the highest for our proposed model.

**TABLE 5:** Performance of the proposed model on the IMDB dataset.

Methods	Accuracy	Precision	Recall	F1-measure
DT	73.78	71.40	79.34	75.16
SVM	86.18	84.62	88.45	86.49
LSTM	82.5	83	79.9	81.3
CNN	87.1	86.8	88.7	87.5
CNN-LSTM	87.9	87.4	88.6	87.7
RoBERTa-LSTM	92.96	93	93	93
RoBERTa-GRU	94.63	95	95	95
Proposed model	96.28	97	97	97

**FIGURE 4:**
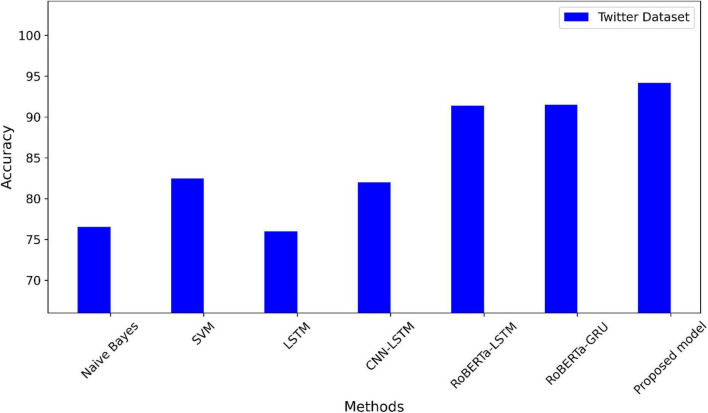
A comparison of the Twitter dataset’s accuracy.

**FIGURE 5:**
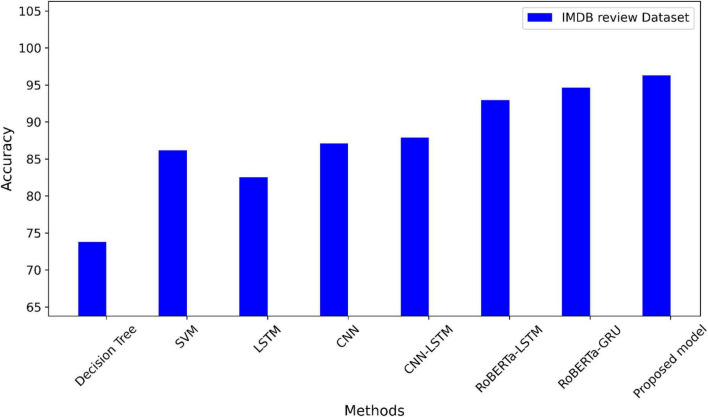
A comparison of the IMDB dataset’s accuracy.

The accuracy of the suggested model is compared to the most advanced techniques using the Twitter dataset in Figure 4. A comparison of the suggested model’s accuracy with other methods using the IMDB dataset is shown in Figure 5. The highest accuracy values in the two datasets using the suggested model.

## 5 Discussion

During this section, we will analyze the experimental results from the previous section. We used real datasets to implement our proposed method. According to our findings, the proposed method performed well, with high accuracy and balanced F1-measure, recall, and precision, which means the model can generalize well to any class. The 256 LSTM units and 256 CNN units, the Adam optimizer, and a learning rate of 0.00001 are the best hyperparameter values for the proposed model after many experiments. The best performance, indicating the ability to capture long-range dependencies, is shown by the 256-unit LSTM layer and 256-unit CNN layer. In addition, when it comes to gradient optimization, the Adam optimizer performs better. A crucial parameter in model training is the learning rate. With a learning rate of 0.0001, the model can converge at the right rate and perform at its best. The early stopping mechanism is applied to the validation accuracy with patience set to 10 epochs to stop the overfitting problem. Table 4 shows that the RoBERTa-(CNN+LSTM) demonstrated significant performance improvements on a dataset with a Twitter dataset. There has been an increase in F1 measures from 79 to 93%. Compared with the competitive methods, there is an improvement in accuracy. A further benefit of using the SMOTE technique was the improvement of accuracy because this technique can address imbalanced classes in the Twitter dataset. It is observed among all models, the proposed model in the Twitter dataset had the highest accuracy, with 94.2%. In terms of accuracy and overall evaluation metrics.

On the IMDb dataset, Table 5 compares the methods with our proposed model. Based on the results presented in this table, there were differing levels of performance in sentiment analysis. RoBERTa-LSTM and RoBERTa-GRU models, which incorporate advanced architectures like RoBERTa, outperform traditional models like DT and SVM. The suggested RoBERTa-(CNN-LSTM) model yields a higher F1-score of 97% than previous studies. The performance of the models has been improved from 73.46 to 96.28% in terms of accuracy in our proposed model.

Overall, by combining transformer-based architectures with hybrid combinations, the proposed model achieves strong performance in sentiment analysis. The text sequence is tokenized and encoded in word embeddings representation with a remarkable performance from the RoBERTa model. The CNN model uses max-pooling and convolutional layers to effectively extract higher-level features. Word sequences with long-term dependencies can be captured by the LSTM model. The strengths of LSTM, CNN, and RoBERTa are combined in the suggested RoBERTa-(CNN+LSTM)model. It is clear from these results that transformer models and hybrid approaches are effective in sentiment classification.

Business organizations may be better equipped to identify negative feedback as a result of the improvements in the sentiment analysis process. Business organizations that examine negative reviews can quickly ascertain the needs of their customers and adjust their policies and products, such as by quickly identifying negative reviews. This proactive strategy can help retain current clients, foster loyalty, and improve the overall customer experience. It also shows attention to meeting customer needs. Some ways in which public opinion can influence government decisions and policy development such as to determine public opinion on issues, governments frequently turn to surveys and opinion polls. These resources can help politicians make decisions by giving them insights into the preferences of the public.

A limitation of this study is that it uses only English datasets, and the training of the suggested model can be computationally expensive, requiring significant training times and high-performance computing resources because they typically require multiple layers, attention mechanisms, convolutions, or recurrent connections.

## 6 Conclusion

In this paper, we suggest a model for sentiment analysis based on two English datasets. The use of a transformer for sentiment analysis is limited. So, the proposed framework is based on a transformer called the RoBERTa and the usage of hybrid learning-based deep neural networks, namely LSTM and CNN, which combine a model called the RoBERTa-(CNN+LSTM). Our proposed model was implemented using real datasets. In the imbalanced Twitter US airlines dataset, we used the SMOTE method for resampling because deep learning models often display a bias in favor of the majority class when the polarity distribution is imbalanced. The model may have trouble correctly predicting the minority class because it has more examples for the majority class to learn from. This bias results from this. Based on our findings, the suggested hybrid model performs best in the datasets using RoBERTa as a word embedding model and achieves a higher level of accuracy than any of the other models. The accuracy of the IMDB movie reviews dataset is 96.28% and in Twitter reviews is 94.2%. RoBERTa model combined with (CNN+LSTM) produces a powerful and efficient sentiment analysis model, making it a promising solution for many NLP tasks. In future studies, other languages, such as Moroccan or Arabic, could be included in the analysis and we can explore other deep learning models that can be merged with other transformer models such as BERT. In addition, we plan to augment data with a Generative pre-trained transformer (GPT-3) as well.

## Data availability statement

Publicly available datasets were analyzed in this study. This data can be found here: https://www.kaggle.com/datasets/crowdflower/twitter-airline-sentiment.

## Author contributions

NS: Writing—original draft, Writing—review and editing. WA: Writing—original draft, Writing—review and editing. KA: Writing—original draft, Writing—review and editing. PP: Writing—original draft, Writing—review and editing. MH: Writing—original draft, Writing—review and editing.

## Acknlowedgements

We would like to acknowledge Prince Sultan University for their valuable support.
